# Socio-demographic, lifestyle and health characteristics among snus users and dual tobacco users in Stockholm County, Sweden

**DOI:** 10.1186/1471-2458-10-619

**Published:** 2010-10-18

**Authors:** Karin Engström, Cecilia Magnusson, Maria Rosaria Galanti

**Affiliations:** 1 Department of Public Health Sciences, Division of Public Health Epidemiology, S-171 76 Stockholm, Sweden

## Abstract

**Background:**

Socio-demographic and lifestyle characteristics of snus users have not been systematically described. Such knowledge is pivotal for tobacco control efforts and for the assessment of health effects of snus use.

**Methods:**

A cross-sectional study was conducted, based on the Stockholm Public Health Survey, including a population-based sample of 34,707 men and women aged 18-84 years. We examined how socio-demographic, lifestyle and health-related characteristics were associated with the prevalence of current daily snus use, smoking and dual tobacco use. Logistic regression was used to calculate odds ratios of prevalence (ORs) and 95% confidence intervals (CIs).

**Results:**

Low educational level (OR = 1.60, CI = 1.41-1.81 and OR = 1.49, CI = 1.17-1.89, for men and women respectively), as well as occupational class and low income were associated with snus use. Some unfavourable lifestyle characteristics, including risky alcohol consumption (males: OR = 1.81, CI = 1.63-2.02; females: OR = 1.79, CI = 1.45-2.20), binge drinking and low consumption of fruit and vegetables were also associated with snus use. In contrast, physical inactivity and overweight/obesity were not, nor was perceived health. The prevalence of smoking followed steeper gradients for social as well as lifestyle characteristics. Overweight and obese men were however less often smokers. Perceived poor general health and psychological distress were highly related to smoking. Social disadvantage, as well as unhealthy lifestyle and self-reported poor health were strongly associated with dual use. There were limited differences between men and women.

**Conclusions:**

The social, lifestyle and health profiles of exclusive snus users in Stockholm County are less favourable than those of non-users of tobacco, but more advantageous than those of exclusive smokers. This knowledge should guide tobacco control measures as well as the interpretation of health risks linked to snus use.

## Background

Tobacco use is the most important contributor to morbidity and premature death among modifiable life style factors [1]. Sweden is the only industrialized country that reached the World Health Organization year 2000 goal of less than 20% adult smokers. However, the pattern of tobacco use in Sweden is unique with a large proportion of the foremost male population being users of a moist smokeless tobacco product called snus. The prevalence of daily smoking among Swedish adults, 16-84 years old, is 13% among men and 15% among women. Corresponding rates for snus use are 19% and 4% [2], with a well-known North to South gradient of decreasing prevalence [3,4].

Snus is a moist, smokeless tobacco product consisting of ground tobacco, water, salt, humectants and flavours [5]. It has been used in Sweden for several centuries and Sweden is the only member state that has been granted an exception from the ban on manufacturing and selling snus within the European Union.

Whether the relatively low prevalence of smoking in Sweden is a consequence of the widespread use of snus is much debated [3,6-13]. There is, however, increasing interest in snus both in Europe and in the USA. The Scientific Committee on Emerging and Newly Identified Health Risks (SCENIHR) - an independent advisory committee serving the European Commission - recently reviewed the health effects of snus and other smokeless tobacco products (STPs) in response to current claims that using snus could reduce population-level harm related to smoking. They concluded that STP use is harmful to health and that the evidence of the effectiveness of STP as a smoking cessation aid is insufficient [14]. In the USA, snus is heavily marketed by the tobacco industry, and there were recent congressional hearings prompted by U.S. Smokeless Tobacco Company requesting permission to make health claims that smokeless tobacco is safer than cigarettes. The scant available evidence on the influence of snus use on risk for cardiovascular disease does not support any overall association, although data from one large cohort study indicate increased risks for fatal myocardial infarction and stroke [14-18]. Available evidence does not support any influence of snus use on risk for oral cancer [19-22] or oropharyngeal cancer [20], while results regarding oesophageal and pancreatic cancer point toward a weak but causal association [19,20].

Whereas the characteristics of smokers are extensively studied, systematic descriptions of social and behavioural profiles of snus users are lacking. Such knowledge is pivotal for effective tobacco control and identification of priority groups for targeted interventions. Furthermore, an understanding of the pattern of tobacco use according to disease risk factors is important for the evaluation of confounding in studies on health effects of snus. Thus, we report on patterns of snus and dual tobacco use according to socio-demographic, lifestyle and health characteristics, using data from a large and recent population-based survey conducted in Stockholm County, Sweden.

## Methods

This is a cross-sectional study using data from self-administered questionnaires, set within The Stockholm Public Health Survey from 2006. Ethical approval for the study was granted by the Stockholm Regional Ethical Review Board.

### Study population and data collection

In all, 57,000 individuals aged 18-84 years were randomly selected from the background population of Stockholm County after stratification for residential municipality. Stockholm County has approximately 1.9 million inhabitants, corresponding to 21% of the Swedish population. Administrative records held by Statistics Sweden, including name, address and date of birth of all Swedish residents, were used to identify the study population [23]. Among invited individuals, 34,707 (61% of all eligible) participated in the survey. Compared with Stockholm county census data, non-respondents included a higher proportion of men, subjects under the age of 45, foreign-born, single or separated, unemployed and in the lowest quartile of income.

Subjects were sent a study invitation via traditional mail, and asked to complete a comprehensive questionnaire, choosing between paper and web-based forms. The latter was available at an URL provided in the letter together with a personal login. Of the respondents, 86.4% chose paper and 13.6% web-based questionnaires. Paper and web-based questionnaires contained identical questions eliciting information on socio-demographic factors, health parameters, physical activity, alcohol consumption, anthropometry and other life circumstances. The majority of items were validated instruments often used for health surveys, in Sweden and elsewhere.

### Measures

Current smoking and snus use was assessed using the following question: "Do you [currently smoke; use snus] daily?". Current tobacco use was categorized into four mutually exclusive groups - no daily use (including former use), exclusive daily use of snus, exclusive daily smoking or daily dual use (both smoking and snus use). Occasional tobacco use was not elicited in the survey and therefore not taken into account in this study.

Age and current profession were reported by the participants while levels of education and income, measured as individualised disposable income calculated from the total family income, were taken from the LISA register maintained by Statistic Sweden [24]. Age was categorized into 10-years intervals. The allocation of occupational class was based on the Swedish Socioeconomic Classification which provides a measure of class based on occupation [25,26]. It classifies individuals in the labour force into self-employed and employed. The latter group is sub-grouped according to the average educational level required for any particular occupation, yielding five occupational categories: unskilled manual worker, skilled manual worker, low-level clerk, intermediate level clerk, high level clerk. Education was grouped in three categories (high: university studies; intermediate: secondary school; low: only compulsory education + vocational training), and income was categorised in quintiles.

Participants were also asked to report their weight and height, their alcohol consumption during an average week (from which a summary variable expressing average consumption in gr of ethanol/week was derived) and their frequency of binge drinking (defined as the equivalent to two bottles of wine during the same occasion). Consumption of fruit and vegetables was assessed by the questions "How often do you eat [fruit or berries; a portion of vegetables or root vegetables]?" Information on physical activity was obtained by asking "How many days per week do you exercise for at least 30 minutes intensely enough to start sweating"? Body Mass Index (BMI) was calculated as the ratio between weight in kg and the square of height in meters (kg/m^2^), and categorised as underweight; normal weight; overweight or obese (corresponding to BMI < 18.0; 18.0-24.9; 25.0-29.9; and ≥ 30). Average alcohol consumption during one week was dichotomized as risky alcohol consumption (men: > = 170 gr pure alcohol/week, equivalent to 14 standard drinks; women: > = 110 gr/week, equivalent to 9 standard drinks) versus not risky, based on the recommendation from the Swedish Council for Information on Alcohol and Other Drugs (CAN). Consumption above the cut-off point is considered to increase the risk of health damages [27]. Three mutually exclusive categories were employed for frequency of binge drinking (less than monthly or never; monthly, but not weekly; weekly). Fruit and vegetable consumption were categorised as once a week or less; several times a week to daily; several times a day. Days per week with at least 30 minutes of intense physical activity were collapsed into three categories (< 2; 2-4; 5+ days/week).

Perceived health was self-reported on a 5-points Likert scale, collapsed into three categories: very poor/poor; fair; very good/good. Lastly, psychological distress was assessed via the twelve-item version of the General Health Questionnaire (GHQ-12). GHQ-12 is a well-validated self-report screening tool that is internationally used to identify general, non-psychotic and context-free mental and affective ill being, so called common mental disorders [28]. The GHQ-12 is also predictive of more severe mental disorders [29]. The score of GHQ-12 ranges from 0 to 12 and the recommended cut-off point of three or more symptoms was employed to define psychological distress [28].

### Statistical analyses

Prevalence odds ratios (ORs) of current daily tobacco use versus no current use were used as measure of cross-sectional associations with selected socio-demographic, lifestyle and health characteristics, based on logistic regression models, using SAS 9.1. In addition, ORs of snus use and dual use versus smoking were calculated with regard to lifestyle and health characteristics. Adjustments were made for age (grouped as 18-44, 45-64, ≥ 65 years) and occupational class, where applicable. Analyses of current daily exclusive snus use or smoking versus no current daily tobacco use were further adjusted for past use of the other type of tobacco. In addition, BMI and physical activity were mutually adjusted, as were education, income and occupational class. All analyses were done separately for men and women.

## Results

Descriptive data on characteristics of the study population are given in Table 1. Overall, 17.0% of males reported to be current exclusive daily snus users, while 2.4% reported combined daily use of snus and cigarettes. Additionally, 11.3% were daily exclusive smokers. Daily smoking was more prevalent among women than men (15.2%). Yet, since only 3.1% of the women were exclusive daily snus users and 0.5% dual users, the total prevalence of current tobacco use was higher among men (30.7%) than among women (18.8%).

**Table 1:** Descriptive characteristics of the Stockholm Public Health Survey 2006.

*Characteristic*	*Males*		*Females*		*All*	
	n	%	n	%	n	%
**Age**						
18-24	1241	7.9	1618	8.5	2859	8.2
25-34	2328	14.9	3293	17.3	5621	16.2
35-44	3086	19.8	3840	20.1	6926	20.0
45-54	2762	17.7	3240	17.0	6002	17.3
55-64	3163	20.2	3423	17.9	6586	19.0
65-74	1963	12.6	2198	11.5	4161	12.0
75-	1080	6.9	1472	7.7	2552	7.4

**Education**						
Low	2720	19.0	3045	17.6	5765	18.2
Intermediate	6115	42.8	7109	41.0	13224	41.8
High	5457	38.2	7174	41.4	12631	39.9

**Disposable income**						
Very low	2829	18.5	3955	21.2	6784	20.0
Low	2821	18.5	3976	21.3	6797	20.1
Intermediate	2919	19.1	3839	20.6	6758	19.9
High	3130	20.5	3649	19.6	6779	20.0
Very High	3559	23.3	3211	17.2	6770	20.0

**Occupational class**						
Unskilled worker	2200	15.8	2973	17.8	5173	16.8
Skilled worker	2166	15.5	1793	10.7	3959	12.9
Low-level clerk	1220	8.7	3470	20.7	4690	15.3
Middle level clerk	3137	22.5	4411	26.3	7548	24.6
High level clerk	3403	24.4	3047	18.2	6450	21.0
Self-employed	1841	13.2	1053	6.3	2894	9.4

**Risky alcohol consumption**						
No	9428	72.4	11001	75.2	20429	73.9
Yes	3586	27.6	3619	24.8	7205	26.1

**Binge drinking**						
Never/seldom	11092	72.8	16764	90.0	27856	82.3
Monthly	2788	18.3	1320	7.1	4108	12.1
Weekly	1357	8.9	545	2.9	1902	5.6

**Fruit consumption**						
Once a week or less	2770	17.9	1394	7.4	4164	12.1
Several times a week/daily	10456	67.5	10848	57.3	21304	61.9
Several times a day	2265	14.6	6680	35.3	8945	26.0

**Vegetable consumption**						
Once a week or less	2439	15.7	1213	6.4	3652	10.6
Several times a week/daily	11273	72.7	12329	65.1	23602	68.5
Several times a day	1784	11.5	5401	28.5	7185	20.9

**Body mass index**						
Underweight	178	1.2	861	4.6	1039	3.1
Normal weight	7127	46.6	11123	60.0	18250	53.9
Overweight	6394	41.8	4696	25.3	11090	32.8
Obese	1608	10.5	5404	10.0	3466	10.2

**Physical activity days/week**						
< 2	4272	28.3	4734	25.9	9006	27.0
2-4	7278	48.1	9231	50.5	16509	49.5
5+	3568	23.6	4298	23.5	7866	23.6

**Self-rated health**						
Very good/good	11330	73.4	13242	70.3	24572	71.7
Fair	3322	21.5	4416	23.4	7738	22.6
Very poor/poor	790	5.1	1179	6.3	1969	5.7

**Psychological distress**						
No	13207	85.6	14847	78.8	28054	81.9
Yes	2213	14.4	3987	21.2	6200	18.1

**Tobacco use**						
No use						
Never use	6057	39.3	9369	49.9	15426	45.1
Former use	3570	23.1	4332	23,1	7902	23,1
Former unknown	1063	6.9	1536	8.2	2599	7.6
Current exclusive snus use	2624	17.0	576	3.1	3200	9.4
Current exclusive smoking	1745	11.3	2853	15.2	4598	13.4
Current dual use	369	2.4	95	0.5	464	1.4

Table 2 provides ORs of tobacco use in relation to socio-demographic characteristics. The prevalence of exclusive snus use was highest in the youngest age categories (below age 35), with ORs declining steadily with increasing age. In contrast, the highest prevalence of smoking was seen in middle age, while the prevalence of dual tobacco use showed two age-related peaks, in age 18-24 and 45-54, for both men and women.

**Table 2:** Cross-sectional odds ratios (OR)^1 ^of current daily tobacco use versus non-use in relation to socio-demographic factors the Stockholm Public Health Survey 2006.

*Characteristic*	*Males*			*Females*		
		
	Snus use OR^2 ^(95% CI)	Smoking OR^3 ^(95% CI)	Dual use OR (95% CI)	Snus use OR^2 ^(95% CI)	Smoking OR^3 ^(95% CI)	Dual use OR (95% CI)
**Age, years**						
18-24	Ref	Ref	Ref	Ref	Ref	Ref
25-34	0.92	1.26	0.64	0.63	0.58	0.66
	(0.74-1.14)	(0.90-1.76)	(0.41-1.01)	(0.43-0.92)	(0.47-0.71)	(0.27-1.63)
35-44	0.85	1.47	0.82	0.72	0.63	0.66
	(0.69-1.06)	(1.06-2.02)	(0.54-1.25)	(0.50-1.04)	(0.51-0.77)	(0.27-1.60)
45-54	0.58	2.00	0.94	0.59	1.02	1.26
	(0.46-0.72)	(1.46-2.74)	(0.62-1.42)	(0.40-0.86)	(0.84-1.24)	(0.55-2.91)
55-64	0.31	2.06	0.61	0.22	0.86	0.80
	(0.25-0.39)	(1.51-2.81)	(0.40-0.94)	(0.15-0.35)	(0.71-1.05)	(0.34-1.92)
65-74	0.18	1.50	0.35	0.14	0.68	0.07
	(0.14-0.24)	(1.07-2.11)	(0.21-0.60)	(0.08-0.25)	(0.55-0.86)	(0.01-0.57)
75+	0.07	0.67	0.02	0.02	0.33	^4^
	(0.05-0.11)	(0.43-1.03)	(0.00-0.16)	(0.00-0.12)	(0.24-0.45)	

**Education**						
Low	1.49	2.36	2.45	1.07	3.23	3.51
	(1.26-1.77)	(1.92-2.91)	(1.68-3.58)	(0.73-1.55)	(2.72-3.84)	(1.68-7.34)
Intermediate	1.60	1.66	1.97	1.49	2.12	2.03
	(1.41-1.81)	(1.39-1.98)	(1.43-2.70)	(1.17-1.89)	(1.84-2.46)	(1.10-3.75)
High	Ref	Ref	Ref	Ref	Ref	Ref

**Disposable income**						
Very low	0.98	1.40	1.28	1.16	1.35	1.16
	(0.82-1.16)	(1.14-1.72)	(0.88-1.86)	(0.82-1.64)	(1.12-1.62)	(0.51-2.62)
Low	1.25	1.15	1.20	1.28	1.14	0.84
	(1.06-1.47)	(0.93-1.42)	(0.83-1.75)	(0.92-1.78)	(0.96-1.37)	(0.36-1.98)
Intermediate	1.25	0.77	0.99	1.29	0.99	0.69
	(1.07-1.46)	(0.62-0.96)	(0.68-1.44)	(0.94-1.78)	(0.82-1.18)	(0.29-1.63)
High	1.24	0.97	1.07	1.17	0.95	1.99
	(1.06-1.43)	(0.79-1.18)	(0.75-1.54)	(0.84-1.62)	(0.79-1.13)	(1.00-3.96)
Very high	Ref	Ref	Ref	Ref	Ref	Ref

**Occupational class**						
Unskilled worker	1.02	2.42	1.95	1.35	2.08	1.28
	(0.85-1.23)	(1.90-3.10)	(1.25-3.04)	(0.92-1.96)	(1.66-2.60)	(0.51-3.22)
Skilled worker	1.40	2.04	2.94	1.30	2.28	1.56
	(1.17-1.68)	(1.59-2.63)	(1.92-4.52)	(0.87-1.94)	(1.80-2.88)	(0.60-4.08)
Low-level clerk	1.14	1.28	1.10	1.00	1.67	1.19
	(0.94-1.40)	(0.95-1.72)	(0.63-1.90)	(0.70-1.43)	(1.34-2.07)	(0.50-2.85)
Middle level clerk	1.06	1.12	1.18	1.27	1.41	1.15
	(0.91-1.24)	(0.88-1.41)	(0.77-1.79)	(0.94-1.73)	(1.15-1.73)	(0.52-2.54)
High level clerk	Ref	Ref	Ref	Ref	Ref	Ref
Self-employed	1.09	1.48	1.32	1.02	1.45	1.09
	(0.90-1.31)	(1.15-1.92)	(0.82-2.13)	(0.63-1.63)	(1.11-1.91)	(0.35-3.41)

^1 ^Adjusted for age, occupational class, disposable income and education, when applicable. ^2 ^Further adjusted for past smoking. ^3 ^Further adjusted for past snus use. ^4^ Model not converging due to small numbers.

Among men, snus use was more prevalent in skilled workers and in individuals with intermediate levels of income (low, intermediate and high income) than in other occupational and income groups. Men with low and intermediate education were more likely to be snus users as compared to men with high educational level. Male smoking behaviour followed a clearer social gradient, with the highest prevalence odds among unskilled workers, individuals with very low income and low educational level. Dual use followed more closely the social patterns of smoking than of exclusive snus use. Among women, snus use was not clearly related to occupational class or income, but was more common in women with intermediate educational level, compared to high. Smoking followed the same socioeconomic gradient seen among men.

ORs of tobacco use in relation to lifestyle and health characteristics are presented in Table 3. Risky total consumption of alcohol as well as binge drinking at least monthly was associated with all kinds of tobacco use among both men and women. The same was true for low consumption of fruit and vegetables, with ORs of tobacco use decreasing with increasing consumption. However, these patterns were more pronounced for smoking and dual tobacco use than for exclusive snus use. For instance, consuming fruits very seldom (once a week or less) was associated with an OR of 2.53 (CI 2.10-3.03) for snus use among men, to be compared with an OR of 5.55 (CI 4.27-7.23) to be a smoker and of 9.63 (CI 5.59-16.6) to be a dual tobacco user. No gradients in snus use with level of physical activity were noted, while a sedentary lifestyle was associated with smoking and dual tobacco use among both genders. Underweight was inversely associated with snus use and positively associated with smoking among men, while overweight was inversely associated with smoking. Among women, BMI was not clearly related to snus use or smoking. The prevalence odds of dual tobacco use were higher among men with overweight, while the association between BMI and dual tobacco use was unclear for women.

**Table 3:** Cross-sectional odds ratios (OR)^1 ^of current daily tobacco use versus non-use in relation to lifestyle and health factors in the Stockholm Public Health Survey 2006.

*Characteristic*	*Males*			*Females*		
		
	Snus use OR^2 ^(95% CI)	Smoking OR^3 ^(95% CI)	Dual use OR (95% CI)	Snus use OR^2 ^(95% CI)	Smoking OR^3 ^(95% CI)	Dual use OR (95% CI)
**Risky alcohol consumption**						
No	Ref	Ref	Ref	Ref	Ref	Ref
Yes	1.81	1.88	2.71	1.79	1.78	3.29
	(1.63-2.02)	(1.62-2.18)	(2.12-3.46)	(1.45-2.20)	(1.58-2.00)	(2.04-5.30)

**Binge drinking**						
Never/seldom	Ref	Ref	Ref	Ref	Ref	Ref
Monthly	2.34	1.82	2.31	1.53	2.80	4.46
	(2.08-2.63)	(1.55-2.14)	(1.75-3.05)	(1.12-2.11)	(2.39-3.27)	(2.50-7.95)
Weekly	3.01	3.16	5.47	3.16	3.85	6.49
	(2.55-3.56)	(2.60-3.84)	(4.07-7.34)	(2.08-4.81)	(3.08-4.82)	(3.02-13.9)

**Fruit consumption**						
Once a week or less	2.53	5.55	9.63	1.80	5.71	4.72
	(2.10-3.03)	(4.27-7.23)	(5.59-16.6)	(1.23-2.64)	(4.78-5.82)	(2.32-9.61)
Several times a week/daily	1.63	2.36	3.06	1.47	2.07	1.52
	(1.39-1.90)	(1.85-3.01)	(1.80-5.20)	(1.19-1.81)	(1.84-2.35)	(0.91-2.55)
Several times a day	Ref	Ref	Ref	Ref	Ref	Ref

**Vegetable consumption**						
Once a week or less	1.71	3.91	5.18	1.36	4.45	2.26
	(1.41-2.07)	(2.97-5.15)	(3.09-8.67)	(0.86-2.15)	(3.64-5.43)	(0.89-5.76)
Several times a week/daily	1.23	1.74	2.01	1.31	1.96	1.44
	(1.05-1.44)	(1.35-2.24)	(1.24-3.28)	(1.06-1.62)	(1.72-2.24)	(0.85-2.42)
Several times a day	Ref	Ref	Ref	Ref	Ref	Ref

**Body mass index^4^**						
Underweight	0.46	1.68	1.27	0.91	1.24	1.76
	(0.22-0.97)	(1.01-2.78)	(0.45-3.59)	(0.55-1.51)	(0.98-1.58)	(0.88-3.52)
Normal weight	Ref	Ref	Ref	Ref	Ref	Ref
Overweight	1.04	0.75	1.30	1.00	1.01	1.63
	(0.93-1.15)	(0.65-0.86)	(1.01-1.66)	(0.79-1.26)	(0.89-1.14)	(0.96-2.76)
Obese	1.01	0.76	1.59	0.99	0.92	1.76
	(0.85-1.20)	(0.61-0.94)	(1.12-2.26)	(0.70-1.39)	(0.77-1.10)	(0.88-3.52)

**Physical activity, days/week^5^**						
< 2	1.09	2.07	1.85	0.90	1.47	1.74
	(0.97-1.22)	(1.79-2.39)	(1.43-2.39)	(0.71-1.14)	(1.31-1.66)	(1.05-2.89)
2-4	Ref	Ref	Ref	Ref	Ref	Ref
5+	0.95	0.94	1.10	1.11	0.99	1.17
	(0.83-1.07)	(0.79-1.12)	(0.82-1.48)	(0.88-1.40)	(0.87-1.13)	(0.65-2.11)

**Self-rated health**						
Very Good/Good	Ref	Ref	Ref	Ref	Ref	Ref
Fair	1.11	1.69	2.16	1.08	1.74	1.44
	(0.98-1.26)	(1.46-1.95)	(1.69-2.77)	(0.86-1.37)	(1.55-1.95)	(0.84-2.48)
Very Poor/Poor	1.11	2.25	2.67	1.02	2.65	3.34
	(0.86-1.42)	(1.76-2.87)	(1.75-4.08)	(0.64-1.64)	(2.22-3.16)	(1.66-6.72)

**Psychosocial distress**						
No	Ref	Ref	Ref	Ref	Ref	Ref
Yes	0.96	1.33	1.68	1.14	1.54	1.63
	(0.83-1.10)	(1.12-1.58)	(1.28-2.20)	(0.91-1.42)	(1.37-1.73)	(1.00-2.66)

^1^Adjusted for age and occupational class, when applicable. ^2^Further adjusted for past smoking. ^3^Further adjusted for past snus use. ^4^Further adjusted for physical activity. ^5^Further adjusted for body mass index.

Perceived poor general health was not associated with snus use. In contrast prevalence odds for smoking as well as dual use, increased with decreasing self-rated health as well as psychological distress, among both men and women.

When comparing prevalence odds for exclusive snus use with those for exclusive smoking (shown in Table 4), low consumption of vegetables and fruit as well as sedentary lifestyle were inversely associated with snus use. There were no appreciable differences between snus use and smoking with regard to risky total consumption of alcohol. However, binge drinking monthly, but not weekly, was positively associated with snus among men, while the reverse was true among women. Both risky consumption and binge drinking were more common among dual users than among smokers. Among men, underweight was inversely and overweight/obesity positively associated with snus use, as compared to smoking. Overweight and obesity were also related to dual use. Among women, underweight was inversely associated with snus use, but no association was seen for overweight and obesity.

**Table 4:** Cross-sectional odds ratios (OR)^1 ^of current daily snus use and dual tobacco use versus smoking in relation to lifestyle and health factors in the Stockholm Public Health Survey 2006.

*Characteristic*	*Males*		*Females*	
		
	Snus use OR (95% CI)	Dual use OR (95% CI)	Snus use OR (95% CI)	Dual use OR (95% CI)
**Risky alcohol consumption**				
No	Ref	Ref	Ref	Ref
Yes	1.06	1.42	1.07	1.79
	(0.91-1.23)	(1.08-1.85)	(0.86-1.33)	(1.10-2.91)

**Binge drinking**				
Never/seldom	Ref	Ref	Ref	Ref
Monthly	1.24	1.20	0.64	1.68
	(1.05-1.47)	(0.89-1.61)	(0.47-0.88)	(0.94-3.02)
Weekly	0.95	1.69	0.77	1.72
	(0.77-1.16)	(1.23-2.32)	(0.51-1.17)	(0.80-3.69)

**Fruit consumption**				
Once a week or less	0.47	1.64	0.36	0.87
	(0.36-0.62)	(0.91-2.96)	(0.25-0.53)	(0.43-1.76)
Several times a week/daily	0.65	1.18	0.76	0.77
	(0.50-0.85)	(0.67-2.10)	(0.61-0.95)	(0.45-1.30)
Several times a day	Ref	Ref	Ref	Ref

**Vegetable consumption**				
Once a week or less	0.41	1.28	0.31	0.57
	(0.30-0.56)	(0.72-2.27)	(0.20-0.49)	(0.22-1.45)
Several times a week/daily	0.63	1.02	0.61	0.72
	(0.48-0.83)	(0.59-1.76)	(0.49-0.76)	(0.43-1.23)
Several times a day	Ref	Ref	Ref	Ref

**Body mass index ^2^**				
Underweight	0.18	0.59	0.51	1.03
	(0.08-0.42)	(0.20-1.78)	(0.30-0.87)	(0.36-2.95)
Normal weight	Ref	Ref	Ref	Ref
Overweight	1.26	1.55	0.97	1.53
	(1.08-1.47)	(1.18-2.04)	(0.77-1.24)	(0.91-2.59)
Obese	1.28	1.89	1.10	1.87
	(1.01-1.63)	(1.28-2.77)	(0.77-1.56)	(0.93-3.74)

**Physical activity, days/week^3^**				
	0.57	0.90	0.60	1.14
< 2	(0.49-0.67)	(0.68-1.20)	(0.47-0.76)	(0.68-1.92)
	Ref	Ref	Ref	Ref
2-4	1.01	1.10	1.10	1.16
5+	(0.84-1.21)	(0.79-1.53)	(0.86-1.41)	(0.64-2.11)

**Self-rated health**				
Very Good/Good	Ref	Ref	Ref	Ref
Fair	0.67	1.19	0.62	^4^
	(0.57-0.79)	(0.91-1.56)	(0.49-0.79)	
Very Poor/Poor	0.50	1.08	0.37	^4^
	(0.37-0.67)	(0.69-1.69)	(0.23-0.60)	

**Psychosocial distress**				
No	Ref	Ref	Ref	Ref
Yes	0.68	1.21	0.77	1.09
	(0.56-0.82)	(0.89-1.64)	(0.61-0.97)	(0.66-1.81)

^1 ^Adjusted for age and occupational class, when applicable. ^2 ^Further adjusted for physical activity. ^3 ^Further adjusted for body mass index. ^4^ Model not converging due to small numbers.

Perceived poor general health and psychological distress according to GHQ12, were inversely associated with snus use as compared to smoking. For instance, the ORs of snus use in individuals reporting psychological distress were 0.50 (CI 0.37-0.67) and 0.37 (CI 0.23-0.60) in men and women respectively.

## Discussion

According to this large and recent population-based survey set in Stockholm County, unfavourable socio-demographic and life style characteristics were associated with snus use. These associations were, however, more pronounced for smoking and dual tobacco use. There was a large and expected [see e.g. [5,8-10,12,30,31]] gender ratio, with snus use being more than 5 times as common among men as among women, while an opposite but weaker ratio was noted for smoking. The age distribution of tobacco use in these data, with snus use and smoking being more common in younger and older individuals respectively, was also expected from prior Swedish studies [see e.g. [4,5,10,32]]. We found indicators of social disadvantage to be less strongly associated with snus use than with smoking. For example, the prevalence of snus use among men with low educational level was 1.5 times the prevalence of those with high educational level, while the prevalence of smoking was more than twice as high among those with low education compared to high. Studies on socioeconomic characteristics of snus users in Sweden [17,32] and Norway [31] are rare but consistent with these findings.

The same pattern was seen for unhealthy lifestyle characteristics. For instance, low consumption of fruit and vegetables was associated with snus use, particularly among men, but to a lower extent than it was with smoking or dual use. Snus use was less than half as likely as smoking among those consuming vegetables and fruit once a week or less. Also, in line with a study from Norway [33], but in contrast to a study from the south of Sweden [34] a sedentary lifestyle was not associated with snus use, while it was clearly associated with smoking and dual use. The prevalence of snus use was 40% lower than smoking among those with a sedentary lifestyle. Differences in the assessments of physical activity may explain this discrepancy between studies. Risky alcohol consumption and binge-drinking, on the other hand, were strongly associated with both snus use and smoking, and especially so with dual tobacco use. This finding is in line with those of previous studies of Swedish adults [16] and adolescents [35,36], as well as those of young adults in Norway [33].

Analyses of tobacco use in relation to health-related characteristics revealed inconstant and rather surprising patterns. Notably, underweight was inversely associated with snus use while the opposite was true for smoking. In addition, smoking was less common among overweight and obese individuals while snus use was not related to overweight. Similar findings have been reported from some [37-39], but not all prior studies [34,40,41]. Although the relation of BMI to snus use is difficult to interpret due to the cross-sectional design, the striking difference with smoking is noteworthy, and should be kept in mind when exploring the health effects of snus use, particularly on cardiovascular and metabolic diseases. Perceived poor general health and psychological distress were not associated with snus use, in contrast with both smoking and dual use. The prevalence of snus use was much lower compared to smoking among those reporting poor general health and psychological distress, especially among women. These findings are partly in line with the scant literature in this area [32,33,42]. Data from the Swedish Survey of Living Conditions (ULF) from 1988-89 including a random national sample of males aged 16-84 years, showed that snus use did not vary according to self-reported health status [32]. In a US-study, however, smokeless tobacco use was found to be associated with anxiety disorder and specific phobia, but not with any other phobia, mood disorders or depression [42].

In summary, the typical exclusive user of snus in this survey was a young man, skilled manual worker of intermediate education, with good self-perceived general and mental health and lifestyle generally not very different from those of non-tobacco users apart from heavier alcohol consumption and lower fruit and vegetables consumption. In contrast, the typical cigarette smoker could be portrayed as a middle-aged manual worker, with low education and very low income, heavy drinker and low-consumer of fruit and vegetables, with low BMI and sedentary lifestyle in leisure time, perceiving poor general and mental health. In addition, dual users constitute a rather small group, burdened with great social disadvantage, as well as with unhealthier lifestyle and self-reported poorer health than any other population group.

The question may arise whether exclusive consumers of snus represent a subgroup of potential tobacco users with the same liability to tobacco dependence as smokers, but with higher "health consciousness". For instance, in an American study the belief that smokeless tobacco is less harmful to health than cigarettes was associated with a four-fold increased likelihood to try this product [43]. If so, one may speculate that in Sweden the availability of snus may have removed from the market of cigarettes this particular susceptible population [44]. Unfortunately, this and similar questions cannot be answered in the frame of the present study, due to several constraints: the cross sectional design, no measures of propensity to consumption or nicotine dependence, and limited information on other psycho-social characteristics, but above all the one-country setting, where this counterfactual outcome [45] cannot be studied.

### Strengths and limitations

To our knowledge, this is the first study presenting a comprehensive description of health related characteristics of snus users in Sweden. The large size of the sample allowed us to include women in the analysis, while most other studies were restricted to men. Other strengths include the wide range of available information on social and health related characteristics.

Non-participation in this survey (about 39% of the original sample) may have lead to selection bias. Because tobacco users are less likely than non-users to participate in surveys the actual prevalence of tobacco use in this population sample may be underestimated [46]. Likewise, the association of tobacco use with health characteristics may be over- or underestimated if non-participation was also correlated with poor health or social disadvantage. However, it is unlikely that this would happen differentially according of the type of tobacco used. Further, all behavioural characteristics including tobacco use may be affected by misclassification due to imperfect recall or infidelity of self-reports, but again this is unlikely to have occurred differentially for snus users compared to smokers. The generalisability of our results to other populations is limited due to product-specific characteristics and the trends of use of Swedish snus compared to smokeless tobacco used in other parts of the world, e.g. in USA, India or other Eastern countries [6,47]. However, the general conclusion of our study, i.e. that there is a need of a careful analysis of socio-demographic as well as lifestyle and health-related characteristics of smokeless tobacco users, is certainly applicable to other contexts, especially when new consumers are likely to emerge in response to a changing market.

## Conclusion

The social, lifestyle and health profiles of exclusive snus users in Stockholm County are more unfavourable compared to non-users of tobacco, but less so than those of exclusive smokers. This finding should guide the interpretation of health risks linked to snus use, particularly in comparison to smokers. In indicated prevention programs, attention should be devoted to the minority group of dual tobacco users, whose characteristics suggest social disadvantage and high frequency of co-morbidity, either related or unrelated to tobacco use.

## Competing interests

The authors declare that they have no competing interests.

## Authors' contributions

KE has conducted the final analyses and wrote the main part of the manuscript. CM conceived the original idea of the study together with MRG, took part in the writing process and contributed importantly to the interpretation of the findings. MRG drafted the analyses plan and the manuscript, conducted a considerable part of the analyses, took part in the writing process and contributed importantly to the discussion of the results. All authors read and approved the final manuscript.

## Pre-publication history

The pre-publication history for this paper can be accessed here:

http://www.biomedcentral.com/1471-2458/10/619/prepub
